# Towards Water, Sodium Chloride and Natural Organic Matter Recovery from Ion Exchange Spent Brine

**DOI:** 10.3390/membranes11040262

**Published:** 2021-04-05

**Authors:** Maryam Haddad, Laurent Bazinet, Benoit Barbeau

**Affiliations:** 1 Department of Chemical Engineering, California State University, Long Beach, CA 90840, USA; 2 NSERC-Industrial Chair on Electromembrane Processes Aiming the Ecoefficiency Improvement of Biofood Production Lines, Department of Food Sciences and Laboratory of Food Processing and ElectroMembrane Processes (LTAPEM), Universite Laval, Quebec, QC G1V 0A6, Canada; laurent.bazinet@fsaa.ulaval.ca; 3 NSERC-Industrial Chair on Drinking Water, Department of Civil, Geological and Mining Engineering (CGM), Polytechnique de Montréal, Montreal, QC H3T 1J4, Canada; benoit.barbeau@polymtl.ca

**Keywords:** spent brine, ion exchange, resource recovery, monovalent selective electrodialysis (MSED), DCMD, stack configuration

## Abstract

Despite the tremendous success of the application of anion exchange resins (IX) in natural organic matter (NOM) removal over conventional removal methods, the considerable amount of brine spent during its regeneration cycle makes its sustainability questionable. This polluting saline stream can be challenging to manage and costly to discharge. Alternatively, and with the recent shift in perception of resource recovery, the produced spent brine can no longer be seen as a polluting waste but as an unconventional source of water, minerals and nutrients. In this research, for the first time, we evaluated the effectiveness of an integrated monovalent selective electrodialysis (MSED) and direct contact membrane distillation (DCMD) system in IX spent brine desalination and resource recovery. Of particular interest were the effects of operating time on the characteristics of the monovalent permselective ion exchange membranes, the impact of the DCMD stack configuration on minimizing heat loss to the ambient environment and the efficacy of the recovered NaCl in the regenerating cycle of the exhausted IXs. Our findings demonstrated that although the recovered NaCl from the stand-alone MSED can restore nearly 60% ion exchange capacity of the exhausted IXs, coupling MSED with DCMD led to minimizing the consumption of fresh NaCl (in the IX regeneration cycle) significantly, the potential application of NOM in agriculture and diminishing the risk of the IX spent brine disposal. In addition, the initial characteristics of the ion permselective membranes were maintained after 24 h of MSED and the transmembrane flux was increased when the feed/hot compartment (in the DCMD stack) was encapsulated on two outer ends with coolant/permeate compartments as a result of less heat loss to the ambient environment.

## 1. Introduction

The presence of natural organic matter (NOM) in drinking water can adversely impact water quality by causing aesthetic issues such as changes in the color, taste and odor. Although exposure to NOM in the environment is not linked to direct health effects, NOM should be removed during the water treatment processes as its presence in drinking water promotes the formation of disinfection by-products and the development of biofilms in distribution systems [1,2].

The removal of NOM from drinking water using ion-exchange resins (IX) has received increased attentions over the last two decades. The removal is based on the exchange of anions (typically chloride sorbed on polyacrylic or polystyrenic resins) with negatively charged NOM molecules [3]. Despite the fact that IX has shown promise and superior performance compared to the conventional NOM removal methods, its sustainable efficacy is directly affected by our ability to properly manage and treat the IX brine produced during regeneration, a goal that has not yet been reached [1]. The composition of the IX spent brine (i.e., containing a high level of NaCl salt and NOM) cannot comply with increasingly restrictive regulations. On the other hand, inexpensive discharge to open or underground water bodies is no longer an option in most countries [4]. In addition, the frequent transportation of the NaCl salt to water treatment facilities is an important constraint preventing small and remote communities to adopt IXs as their NOM treatment process.

To date, only a few studies addressed the management of the spent brine generated by an IX process intended for NOM removal. A number of researchers suggested reducing the annual generation of the IX spent brine by recycling and reusing it several times prior to its disposal [5,6,7]. Even though this seems, in theory, to be an inexpensive strategy that can decrease the volume of the IX spent brine and NaCl consumption [5,6,8], the ability of the spent brine to regenerate the exhausted resins would diminish after a few cycles [7]. Thus, this approach is not a sustainable waste management strategy and the treatment of the brine is inevitable. In recent years, a few studies documented the possibility of the safe treatment of the IX spent brine using membrane-based techniques (mainly using nanofiltration) [9,10]. Nonetheless, the severe membrane NOM fouling of the nanofiltration membranes has impaired the long-term applicability of this option [9,10,11].

With waste management shifting from treatment towards valorization and resource recovery in recent years, the desalination of IX spent brine for the purpose of salt, water and NOM recovery is an appealing approach. Ideally, the recovered NaCl would be reused for the IX regeneration step and water and NOM, which mainly contains humic substances, could be used in agricultural fields [12]. To this end, in 2019 two different studies explored the possibility of IX spent brine desalination via electrodialysis (ED) [13,14]. The findings of these efforts revealed that the presence of NOM in the IX spent brine led to the fouling of conventional anion exchange membranes and hindered the overall performance of the process and membrane characteristics [13,14]. In our recent work, we demonstrated that by performing the desalination of the IX spent brine under the pulsed electric field, one can decrease the membrane fouling and intensify the desalination process. In addition, we reported that the ED desalination of the IX spent brine with monovalent ion permselective membranes holds promises [14]. However, further longer-term investigations are required to corroborate the viability of the ED with monovalent ion permselective membranes (or monovalent selective electrodialysis (MSED)) in resource recovery from the IX spent brine. In other words, to propose the desalination of the IX spent brine via MSED as a processing strategy, the impact of a longer MSED run on the overall performance of the process and membrane characteristics needs to be examined. Moreover, the effectiveness of the produced NaCl (and the possibility of a post-concentration process) for the regeneration of the exhausted IX resins has to be determined. In this context, the objectives of the present study were to:

(i) investigate the impact of the process intensification on the overall performance of the MSED desalination of the IX spent brine and membrane characteristics,
(ii) assess the feasibility of the further concentration of produced NaCl by integrating the MSED step with a modified direct contact membrane distillation (DCMD) process in which the amount of heat loss to the ambient environment can be reduced,
(iii) examine the efficiency of the recovered NaCl (from MSED and MSED-DCMD processes) in the regeneration of exhausted IXs.

The outcome of this investigation gives a clear and general insight into the influence of the DCMD stack configuration on minimizing the heat loss and improving the process efficiency, a factor that has gained less attention in previous studies. Furthermore, the end results of this work enable us to assess the viability of implementing the integrated MSED-DCMD desalination process, which can result in minimal NaCl consumption, the potential application of NOM in agriculture and lessening the risk of the IX spent brine disposal.

## 2. Experimental

### 2.1. Membranes, Resins and Chemicals

The membranes used in this study were the Neosepta monovalent permselective ion exchange membranes (IEMs) (i.e., ACS/CMS) supplied by ASTOM (Tokyo, Japan) and hydrophobic Polyvinylidene Fluoride (PVDF) purchased from Millipore-Sigma™(DVPP14250, Burlington, NJ, USA). The IX spent brine was collected from the regeneration step a pilot IX column placed at Pont-Viau water treatment plant (Laval, QC, Canada), which is filled with anion exchange A860 resins (Purolite, PA, USA). This column is used to remove NOM from surface water. The column regeneration took place after 7 days of operation (336-bed volumes, BV) with a fresh NaCl (10 wt.%) solution. The NaCl and NOM contents (reported as dissolved organic carbon) of the IX spent brine were 90 g/L and 1 g C/L, respectively. Analytical grade salts and solutions (i.e., NaCl, Na${}_{2}$SO${}_{4}$, NaNO${}_{3}$, K${}_{2}$CrO${}_{4}$, AgNO${}_{3}$) were purchased from VWR International (Pennsylvania, USA), and ultra-pure water was used to prepare aqueous solutions.

### 2.2. Protocol

The experimental assays were conducted in three different phases, (1) short and long runs of monovalent selective electrodialysis (MSED) of the IX spent brine, (2) concentration of the recovered NaCl solution from the MSED step with a modified direct contact membrane distillation (DCMD) and (3) IX regeneration. The details of each phase are described in the following:

#### 2.2.1. MSED

**Figure 1:**
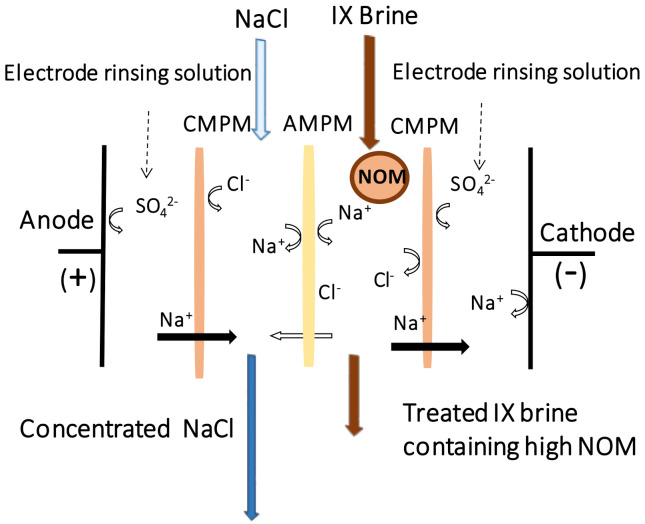
A schematic illustration of the MSED stack used for the IX spent brine desalination.

Two series of short-run (i.e., 3 h) and long-run (i.e., 24 h) MSED experiments were conducted to study the effect of process intensification on the membrane durability, quality of the produced NaCl and overall performance of the desalination process. Each run included 3 consecutive replicate assays carried out at room temperature using a lab-scale ED cell (Micro Flow Cell, ElectroCel, Denmark). As illustrated in (Figure 1), inside the cell cation monovalent permselective membranes (CMPM) and monovalent permselective membranes (AMPM) were encapsulated at one end by an anode compartment and at the other end by a cathode compartment containing the electrode rinsing solution (20 g/L Na${}_{2}$SO${}_{4}$). Throughout each MSED run, applied voltage and current variation along with the electrical conductivity, pH and temperature of each compartment were monitored and recorded. The main operating conditions are presented in Appendix A.

#### 2.2.2. DCMD

**Figure 2:**
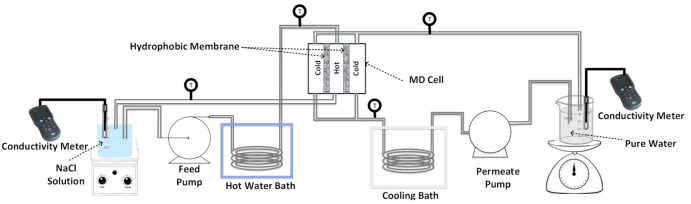
Diagram of the DCMD experimental apparatus.

**Figure 3:**
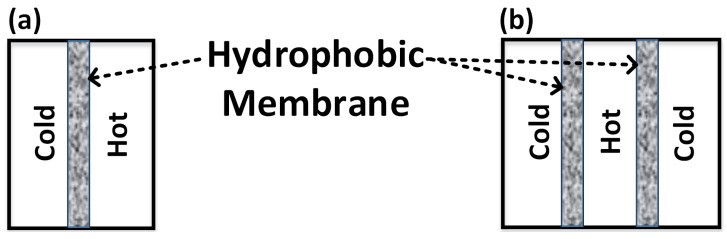
DCMD (**a**) conventional and (**b**) modified stack configurations.

The lab-scale flat sheet DCMD apparatus used for this study was locally designed and fabricated. A schematic illustration of the set-up is presented in Figure 2. In each experiment, the NaCl/feed solution was preheated to the desired temperature and circulated through the feed side of the membrane module and ultra-pure water was circulated through the other side of the module simultaneously in a counter-current mode. The feed and permeate temperatures were adjusted and controlled using thermo-regulators. The weight, conductivity and pH of each compartment as well as the temperature of the inflow and outflow tubes were monitored and recorded frequently. Two different DCMD cell configurations were tested. In conventional configuration (Figure 3a), a single flat sheet PVDF membrane (with an effective area of 10 cm${}^{2}$) was sandwiched between the hot and cold compartments. For the second configuration (Figure 3b) we aimed to minimize the heat loss to the ambient environment by placing the hot compartment in the middle and separating it from the two outer cold compartments by two flat sheet PVDF membranes (with a total effective area of 20 cm${}^{2}$).

The goal of the first set of DCMD tests was to investigate the influence of the DCMD cell configuration and feed temperature. Four experiments were carried out at various feed inlet temperatures of 60 ${}^{\circ }$C, 70 ${}^{\circ }$C, 80 ${}^{\circ }$C and 90 ${}^{\circ }$C, using recovered NaCl solution from the MSED step as well as a synthetic NaCl solution (with similar salt concentration) as the control case. The flow rate of both feed and permeate compartments were set at 1 L/min. Based on the findings of the first set of the assays, the impact of four different feed flow rates on the overall performance of the process was studied. For all the DCMD experiments, the temperature of the cold compartment was maintained at 15 ± 1 ${}^{\circ }$C.

#### 2.2.3. Ion-Exchange Resin Regeneration

The ion-exchange reversible reaction(s) between the negatively charged resins and solution can be written as:

(1) $$n\left[R^{+}\right]Cl^{-}+NOM^{n-}⇋n\left[R^{+}\right]NOM^{n-}+Cl^{-}$$

where *R* is the resin functional group. As one can see, the forward reaction can represent the NOM removal from water by the IXs whereas as the backward reaction demonstrates the regeneration of the exhausted IXs [1].

To perform the regeneration cycle, a sample of exhausted IX resins was collected from the Pont-Viau IX column after 7 consecutive days of operation. To regenerate the resins, an aliquot of resins was added to a 3-bed volume of NaCl solution and agitated for 30 min at 300 rpm. Then, the resins were filtered out and rinsed with a 3-bed volume of ultra-pure water. The ion exchange capacity of the resins before and after the regeneration cycle was measured by titration. The efficacies of three different NaCl solutions was compared in this step: (i) synthetic NaCl solution with 10 wt.% concentration (ii) recovered NaCl from the MSED step and (iii) concentrated NaCl from the MSED-DCMD process were compared in this step. Three replicate regeneration steps were performed at room temperature.

### 2.3. Analyses

#### 2.3.1. Membrane Characterization

The thickness, contact angle and ion exchange capacity of the virgin and used monovalent permselective ion exchange membranes were measured according to the procedures described in [14]. Likewise, the thickness and contact angle of the fresh and used and hydrophobic PVDF membranes were recorded. In addition, the ion exchange capacities of virgin and used monovalent permselective ion exchange membranes were determined.

#### 2.3.2. Ion-exchange Capacity of  the Resins (IEC)

The anion exchange capacity (expressed as mEq/mL of resins) of fresh, exhausted, and regenerated resins were determined by titration. To perform this measurement, 10 mL of resins were added to 170 mL of NaNO${}_{3}$ (250 g/L) and agitated for 30 min at 190 rpm to replace Cl${}^{-}$ by NO${}_{3}^{-}$. Then the resins were filtered out and 15 mL of the filtrate was spiked with 1 mL of K${}_{2}$CrO${}_{4}$ (20 g/L) and titrated with 0.4 N AgNO${}_{3}$ until the AgCl precipitation took place. At this point, the color of the solution would turn orange.

#### 2.3.3. Analytical Measurements

NOM (quantified as total dissolved carbon (DOC)) Content: The DOC content of the IX brine and NaCl solutions as well as the recovered water in the DCMD step were measured at room temperature using a total organic carbon analyzer (M5310C, GE Instruments, USA) with an operating range of 4 ppb to 50 ppm, precision of <1% RSD and accuracy of ±2% or ±0.5 ppb.

Sodium, Chlorine, Bicarbonate and Sulfate Concentrations: The sodium concentration of the brine, NaCl and water samples was measured using an inductively coupled plasma atomic emission spectroscopy (ICP-OES Agilent 5110 SVDV Agilent Technologies, Victoria, Australia) using 588.995 and 589.592 wavelengths. The chloride and sulfate levels were measured via the ion chromatography (Quikchem 8500 series 2, Zellweger Analytic, Inc., Lachat Instruments Division, Milwaukee, WI, USA) applying the Quikchem method 10-117-07-1-C method. An alkalinity method (SM 23 2320-B) was applied to calculate the bicarbonate content of the brine, NaCl and water samples.

#### 2.3.4. Global System Resistance

Ohm’s law was used to calculate the global system resistance:

(2) $$R=\frac{U}{I}$$

where *R* is the global system resistance (Ω), *I* is the current (A) and *U* represents the applied voltage (V) across the MSED stack.

#### 2.3.5. Degree of Desalination

The desalination degree of the IX spent brine was determined as:

(3) $$D_{IXspentbrine}=\frac{{\left[Na\right]}_{i}-{\left[Na\right]}_{f}}{{\left[Na\right]}_{i}}\times 100$$

where ${\left[Na\right]}_{i}$ and ${\left[Na\right]}_{f}$ show the initial and final sodium level (g/L) of the IX spent brine, respectively.

#### 2.3.6. Statistical Analyses

The experimental results were reported as means ± standard deviation and treated by one-way, or if needed multiple-way, statistical analyses of variance with setting the common level of significance, α, at =0.05.

## 3. Results and Discussion

### 3.1. Impact of MSED Process Intensification

**Figure 4:**
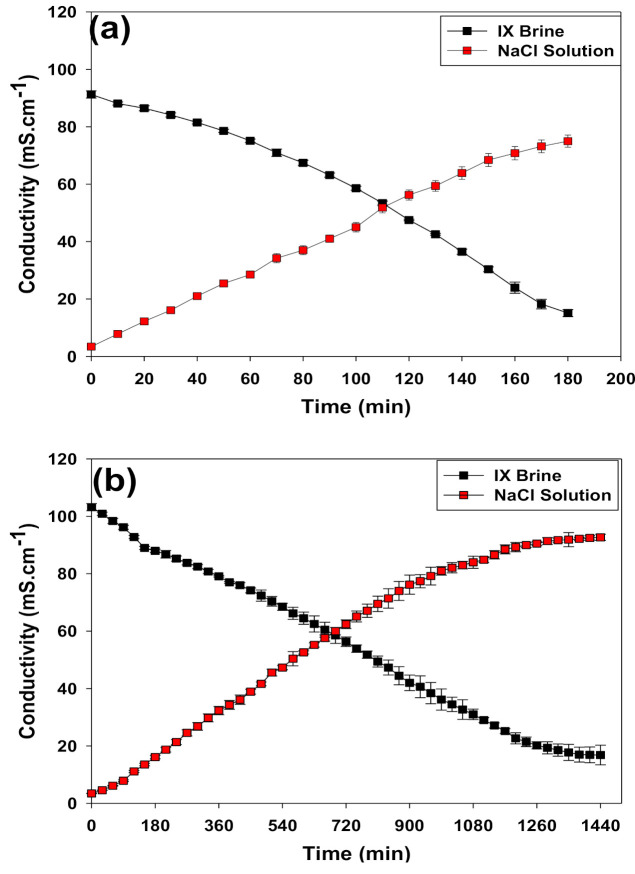
Evolutions of the IX spent brine and NaCl conductivity trends during the (**a**) short runs and (**b**) long runs of MSED.

The evolutions of the IX spent brine and NaCl conductivity profiles are shown in Figure 4. Clearly, there is no significant difference in the behaviour of the IX spent brine and NaCl conductivity profiles were detected once we prolonged the desalination process and ran it for 24 h. For both short and long MSED runs, the conductivity of the spent IX brine decreases linearly over time (0.96 ≤ $R^{2}$ ≤ 0.98) as a result of the transfer of Na${}^{+}$ and Cl${}^{-}$ ions to the concentrate (i.e., NaCl) compartment. Accordingly, due to the increase in the ion concentration in the NaCl compartment, its conductivity starts to rise linearly (0.95 ≤ $R^{2}$ ≤ 0.98) during the MSED desalination process.

**Figure 5:**
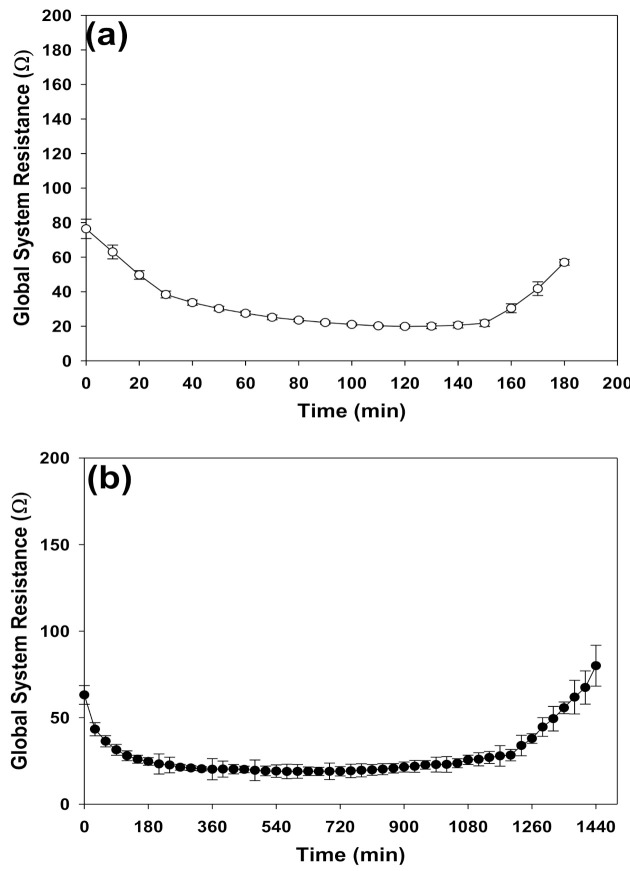
Global system resistance profiles: (**a**) short runs and (**b**) long runs of the IX spent brine desalination via MSED.

Similar to the conductivity profiles, the global system resistance for the short (i.e., 3 h) and long MSED (i.e., 24 h) runs exhibited similar “U” shape trends (Figure 5). The initial sharp decrease in the global system resistance is attributed to the ion transfer from the IX spent brine to the concentrate compartment once the electrical field was applied. By continuing the MSED desalination process, the system reached a steady-state condition and no considerable change in the global system resistance took place [15]. However, towards the end of the desalination process the global system resistance started to rise. Generally, in the absence of membrane fouling, this increase can be attributed to the depletion of the salts in the dilute compartment [15].

Based on the data presented in Table 1, one can note that the degree of the desalination of the IX spent brine for the long run MSED was slightly higher than the degree of the desalination of the IX spent brine when the MSED process was run for only 3 h. The purity of the NaCl solution in terms of the DOC and sulfate levels did not change significantly (α > 0.06) when switching from a 3 h to 24 h operation of the MSED process. It is worth mentioning that the evolution of the conductivity can increase the global system resistance. In short runs, the global system resistance started to increase when the desalination degree reached 89%, while with long runs we saw an increase in the global system resistance around when the desalination degree was close to 92%. Similarly, at these desalination degrees the evolution of the conductivity profiles slowed down (Figure 4) which can be related to the osmotic water transport from the IX spent brine to NaCl compartments [16,17]. The transport of osmotic water occurs mostly when the difference in the concentration of the dilute and concentrated compartments becomes significant [18].

The main specifications of the virgin and used monovalent permselective ion exchange membranes were checked before and after each MSED run. Regardless of the operational time, no formed layer was visually observed on the surface of the monovalent permselective ion exchange membranes. While the characteristics of the CMS membranes remained the same after each MSED experiment (data not presented), the presence of NOM in the IX brine did not significantly impact the initial characteristics of the ACS membranes independent of the operation period (Table 2). These findings are in line with our previous results reporting the characteristics of ACS and CMS membranes under direct and pulsed electric fields [14]. Given the fact that no detectable fouled layer was observed on the surface of the membranes and the initial characteristics of the membranes were maintained (Table 2), we can presume that the increase in the global system resistance and slowing down of the highly concentrated NaCl production was due to the transport of the osmotic water in the opposite direction [16].

### 3.2. DCMD Performance

**Figure 6:**
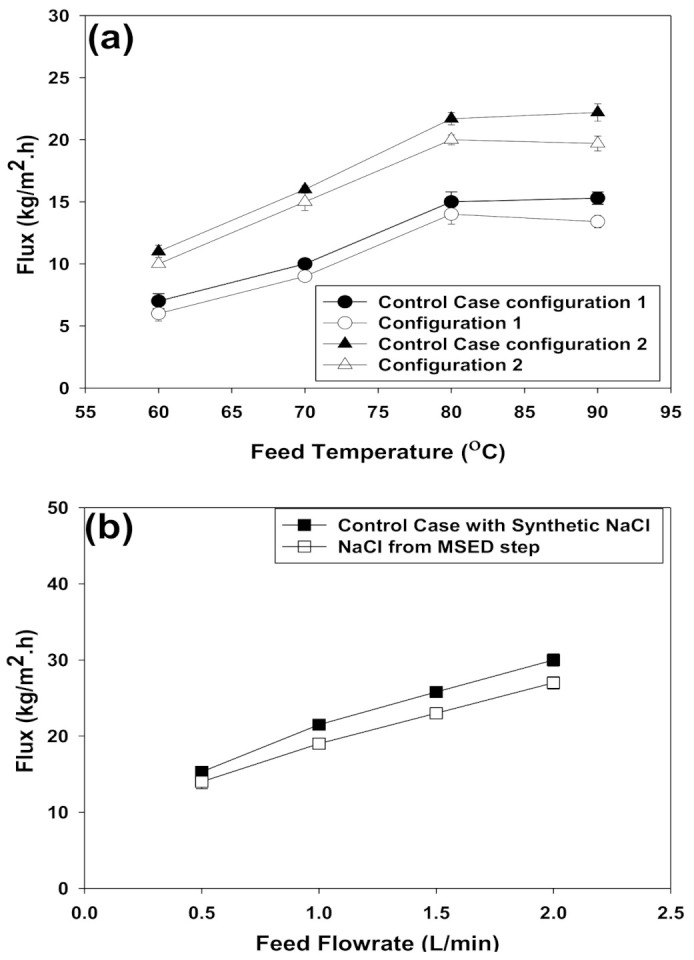
Transmembrane flux as a function of (**a**) feed temperature, (**b**) feed flow rate.

Figure 6a illustrates the impact of the feed temperature and stack configuration on the transmembrane flux during the DCMD process using recovered NaCl solution from the MSED process or synthetic NaCl solution as the control case. For all the tested conditions, increasing the feed temperature from 60 ${}^{\circ }$C to 80 ${}^{\circ }$C led to an increase in the transmembrane flux; however, increasing the feed temperature to 90 ${}^{\circ }$C did not enhance the transmembrane flux. Moreover, significantly higher transmembrane fluxes were achieved when the modified DCMD stack configuration was tested (α=0.02). Indeed, the driving force of a DCMD process is the difference in vapor pressure across the membrane; thus rising the feed temperature induced the vapor pressure in the feed compartment and, consequently, a higher transmembrane flux was achieved when the feed temperature was increased from 60 ${}^{\circ }$C to 80 ${}^{\circ }$C. On the other hand, previous studies indicated that the impact of the temperature polarization phenomenon becomes more pronounced at higher feed temperatures (≥90 ${}^{\circ }$C) [19,20,21], which can adversely affect membrane selectivity and cause membrane wetting [21]. By comparing the contact angle data of the virgin and used PVDF membranes at 90 ${}^{\circ }$C in Table 3, it is apparent that membrane wetting took place which adversely impacted membrane characteristics and particularly membrane hydrophobicity. Conversely, no significant change in the contact angles of the used membranes was detected at the operating temperature range of 60–80 ${}^{\circ }$C (data not presented). In Appendix B, photographs of the used PVDF membranes after conducting the DCMD assays at 90 ${}^{\circ }$C feed temperature illustrate the membrane wetting phenomenon.

In terms of the DCMD stack configuration, sandwiching the heat/feed compartment in between two cold/permeate compartments improved the mass transfer from both sides and lessened the heat loss to the ambient environment; accordingly, higher transmembrane fluxes were obtained. Schwantes and co-workers have also reported that placing the cold channels at the outer sided decreased the heat loss in the feed air gap membrane distillation process [22].

As expected, enhancing the feed flow rate had a direct influence on the transmembrane flux (Figure 6b) and no significant difference between the control case (i.e., using synthetic NaCl solution as the feed) and the actual case (when recovered NaCl from the MSED step was used as the feed) was observed (α=0.07). Note that, based on the findings of the effect of feed temperature and DCMD stack configuration, this series of experiments was carried out at 80 ${}^{\circ }$C using the modified stack configuration. Achieving a higher transmembrane flux as a result of rising the feed flow rate is attributed to the higher turbulence inside the feed channel and the decrease in temperature and concentration polarizations [20]. As Khalifa and co-workers demonstrated, increasing the feed flow rate would promote the mixing phenomenon in the boundary layer next to the membrane which, subsequently, would result in higher heat and mass transfer coefficients [20].

In Table 4, the efficacy of the DCMD process in concentrating the recovered NaCl from the MSED step as well as its impurity level in terms of the DOC and sulfate contents are given. As one can note, after only 3 h of the lab-scale DCMD operation the sodium content of the recovered NaCl from the MSED step increased by more than 2 folds. A low trace of sodium was detected in water samples collected from the permeate side after the DCDM tests while no DOC or sulfate was detected in the permeate compartment. Given the fact that no obvious membrane wetting was spotted under the applied operation conditions (feed temperature: 80 ${}^{\circ }$C, feed flow rate: 2 L/min and modified stack configuration), we can presume that prolonging the duration of the DCMD step would lead to a higher concentration of NaCl; however, further investigations are required to optimize the best operational period and conditions in terms of process productivity and energy consumption.

### 3.3. Resin Regeneration

**Figure 7:**
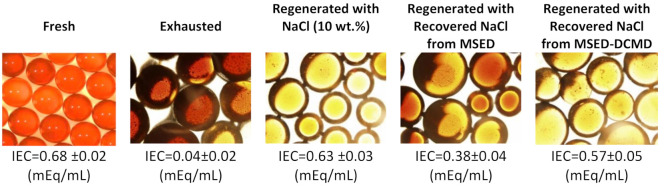
Microscopic images of the fresh, exhausted and regenerated IX resins used in the NOM removal process (scale: 500 μm). (The image of the fresh resins was provided by its supplier).

The efficacies of the 10 wt.% NaCl solution, the recovered NaCl from the MSED step, or the concentrated NaCl from the MSED-DCMD process in regenerating the exhausted IX resins were determined by measuring the IEC of the resins before and after the regeneration steps. As depicted in Figure 7, after a week of operation, the IEC of the exhausted resins dropped significantly (α= 0.03) and only 6% of the chloride exchange capacity of the IXs was still available. Regeneration of the exhausted resins with 10 wt.% NaCl solution could considerably restore the IEC of the resins (α= 0.04). When recovered NaCl from the MSED desalination process was used, more than 55% of the IEC of the exhausted resins was resumed. While up to 85% of the IEC of the exhausted IXs was achieved once we used the concentrated NaCl solution from the integrated MSED-DCMD process.

Based on the findings of the IXs regeneration step, we can deduce that the recovered NaCl from the IX spent brine desalination via MSED can be used in the regeneration cycle of the IX resins. However, to restore the maximum IEC of the IXs one can (i) perform a more frequent regeneration cycle (for example 2–3 time/week) or (ii) add a small amount of fresh NaCl salt to the recovered NaCl solution and increase its concentration. As an alternative and to minimize the amount of salt consumption, the MSED desalination process can be coupled with a DCMD step for further concentration of the NaCl solution. Although our experimental results demonstrated that with a slight modification of the regeneration cycle (e.g., conducting a longer period), the integrated MSED-DCMD desalination method can lead to a significant decrease of the fresh NaCl consumption and minimal risk of the IX spent brine disposal, further investigations and conducting a life cycle assessment are needed to examine the sustainability of this waste management solution in the pilot and large scales. In addition, the trade-off between increasing the feed temperature and feed flow rate during the DCMD step requires further investigations and should be covered during the sustainability study.

## 4. Conclusions

This study set out to determine the feasibility of eco-efficiently treating of the polluting saline waste generated during the removal of NOM from drinking water resources via IX and assessing the viability of MSED-DCMD for salt recovery from the spent IX brine.To this end, the IX spent brine was treated using a stand-alone MSED process as well as an integrated MSED-DCMD system in order to recover and reuse NaCl solution, water and NOM as the products. The recovered NaCl solution was used to regenerate exhausted IXs, while the salt-free NOM solution and water can potentially be used in agricultural applications. The main findings can be summarized as follows:

- Increasing the operation time of the MSED desalination process by 8-fold did not impair the characteristics of the ion permselective membranes (contact angle, thickness and IEC), no membrane fouling took place and high-purity NaCl solution was produced.
- Coupling the MSED desalination step with a DCMD process resulted in a higher concentration of NaCl and pure water recovery from the permeate line.
- Feed temperature and flow rate along with stack configuration had notable impacts on transmembrane flux during the DCMD process. Increasing the feed temperature from 60 to 80 ${}^{\circ }$C significantly improved the transmembrane flux, while membrane wetting occurred when operating the DCMD at 90 ${}^{\circ }$C. Rising the feed flow rate promoted the transmembrane flux as a result of decreasing the temperature and concentration polarizations. Furthermore, sandwiching the feed/heat compartment with two outer coolant/permeate compartments led to minimizing the heat loss to the ambient environment and, consequently, increasing the transmembrane flux.
- High transmembrane fluxes (22 (kg/m${}^{2}$ h)) were recorded when conducting the short (i.e., 3 h) DCMD test with modified stack configuration at 80 ${}^{\circ }$C feed temperature and 2 L/min feed flow rate.
- More than 55% of the IEC of the exhausted resins was restored once we used the recovered NaCl from the stand-alone MSED desalination process of the IX spent brine; whereas the concentrated NaCl solution from the integrated MSED-DCMD process could recover up to 85% of the IEC of the exhausted IXs.

Future work will need to optimize the DCMD step and IX regeneration cycle in order to minimize the energy and salt consumption. In addition, the sustainability of this waste management and resource recovery approach has to be thoroughly addressed by conducting a life cycle assessment.

## Figures and Tables

**Table 1:** Sodium, DOC and sulfate levels of the dilute and concentrate compartments before and after each MSED run

Solution	Na (mg/L)		DOC (mg/L)		SO${}_{4}^{2-}$ (mg/L)	
	Short Run	Long Run	Short Run	Long Run	Short Run	Long Run
Fresh IX brine	32,008 ± 20	31,903 ± 17	1042 ± 15	1074 ± 11	995 ± 4	1001 ± 6
Treated IX brine	3522 ± 8	2552 ± 13	1083 ± 18	1106 ± 16	1068 ± 5	1080 ± 3
Fresh NaCl	763 ± 7	805 ± 8	0 ± 0	0 ± 0	0 ± 0	0 ± 0
Concentrated NaCl	6944 ± 9	13,420 ± 16	0.30 ± 0.09	0.34 ± 0.12	2 ± 1	3 ± 1

**Table 2:** Measured specifications of the ACS membranes before and after each MSED experiment.

Properties	ACS Short Run		ACS Long Run	
	Virgin	Used	Virgin	Used
Thickness (mm)	0.124 ± 0.002 ${}^{a,}$*	0.125 ± 0.003 ${}^{a}$	0.125 ± 0.003 ${}^{a}$	0.125 ± 0.003 ${}^{a}$
Ion Exchange Capacity (meq/g)	1.45 ± 0.03 ${}^{a,}$*	1.44 ± 0.03 ${}^{a}$	1.46 ± 0.02 ${}^{a}$	1.44 ± 0.03 ${}^{a}$
Contact Angle (${}^{\circ }$)	57.5 ± 2.4 ${}^{a,}$*	56.2 ± 1.9 ${}^{a}$	57.6 ± 2.0 ${}^{a}$	54.6 ± 2.7 ${}^{a}$

* The mean values (presented at each row) for fresh and used membranes followed by the letter “${}^{a}$” indicate no significant difference (α < 0.05).

**Table 3:** Thickness and contact angles of virgin and used PVDF membranes.

Properties	Virgin PVDF	Conventional Stack Configuration	Modified Stack Configuration	
		Used PVDF	Used PVDF 1	Used PVDF 2
Thickness (mm)	0.125 ± 4 ${}^{a,}$*	0.117 ± 7 ${}^{a}$	0.118 ± 5 ${}^{a}$	0.119 ± 4 ${}^{a}$
Contact Angle (${}^{\circ }$)	0.120 ± 3 ${}^{a,}$*	104 ± 10 ${}^{b}$	107 ± 8 ${}^{b}$	106 ± 9 ${}^{b}$

* The mean values (presented at each row) for virgin and used membranes followed by different letters “${}^{a}$” and “${}^{b}$” are significantly different (α < 0.05).

**Table 4:** Sodium, DOC and sulfate levels of the feed and permeate streams before and after DCMD tests using configuration 2—i.e., the modified DCMD stack (feed temperature: 80 ${}^{\circ }$C, feed flow rate: 2 L/min).

Solution	Na mg/L	DOC mg/L	SO${}_{4}^{2-}$ (mg/L)
NaCl from MSED step	13,420 ± 16	0.34 ± 0.12	3 ± 1
Concentrated NaCl after DCMD	29,531 ± 19	0.35 ± 0.15	3 ± 1
Pure water before DCMD	0 ± 0	Not Detected	Not Detected
Pure water after DCMD	14 ± 4	Not Detected	Not Detected

**Figure A1:**
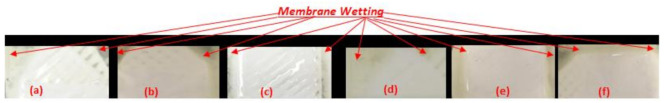
Photographs of used PVDF membranes after DCMD test at 90 ${}^{\circ }$C (**a**) control case configuration (1), (**b**) configuration (1), (**c**,**d**) control cases configuration (2), and (**e**,**f**) configuration (2).

## Data Availability

Not Applicable.

## Appendix A

**Table A1:** Operational conditions of MESD short and long runs.

Operating Conditions	Value
Operational Mode	batch
Number of Operating Units	3
Re-circulation flowrate of each compartment	400 mL min${}^{-1}$
Applied voltage *	12 V
Effective membrane surface area of each membrane	10 cm${}^{2}$
Thickness of each spacer	0.7 mm
Operating temperature	22–23 ${}^{\circ }$C
Initial volume of each compartment in short run	100 mL
Initial volume of each compartment in long run	1000 mL

* The value was corresponding to 75% of the limiting current density measured according to Cowan and Brown procedure [23].
